# Supplementary material for: High throughput morphological screening identifies chemically defined media for mesenchymal stromal cells that enhances proliferation and supports maintenance of immunomodulatory function
Source: Stem Cell Res Ther. 2025 Mar 7;16:125. doi: 10.1186/s13287-025-04206-8 (PMC11889916; doi:10.1186/s13287-025-04206-8)
Supplement: Supplementary file 6 — Supplementary Material 6 [file 13287_2025_4206_MOESM6_ESM.pdf]

Cell-line	Tissue Source	Donor Age	Donor Sex	PDL
Ad98	Adipose	18-30	Female	12
BM71	Bone marrow	18-30	Female	13
BM115	Bone marrow	20	Female	15

Table S1: Donor information.

A)

CHEMICALLY DEFINED BASAL MEDIA (CDBM)				
**Component**	**Company**	**Catalog No.**	**Concentration (ug/mL)**	**Volume Ratio**
AdvancedMEM Basal Media*	Gibco	12492013	-	97.78%
GlutaMAX	Gibco	35050061	-	1.000%
Pen/Strep	Gibco	15140122	-	1.000%
Chemically Defined Lipid Concentrate**	Sigma-Aldrich	L0288	-	0.100%
Putrescine	Sigma-Aldrich	P5780	10	0.100%
Hydrocortisone	Sigma-Aldrich	H0888	0.05	0.008%
Progesterone	Sigma-Aldrich	P8783	0.005	0.010%

*https://www.thermofisher.com/order/catalog/product/12492013

**https://www.sigmaaldrich.com/US/en/product/sigma/l0288?srsltid=AfmBOopYkr4EzlWhiHCnkrpHshQfISV9e6hCNzv3olzRAzLelmTapDKh

B)

SERUM CONTAINING MEDIA (SCM)			
**Component**	**Company**	**Catalog No.**	**Volume Ratio**
αMEM Basal Media	Gibco	12492013	88%
GlutaMAX	Gibco	35050061	1%
Pen/Strep	Gibco	15140122	1%
Fetal Bovine Serum (FBS)	Neuromics	218H19	10%

Table S2: Media formulations for A) chemically defined basal media (CDBM) and B) serum-containing media (SCM). CDM hit formulations are CDBM supplemented with growth factors as defined in main figures. Full formulations for AdvancedMEM Basal Media and Chemically Defined Lipid Concentrate can be found via the links provided.

Figure	Experiment Description	Variables		Controls	
		Independent Variables	Dependent Variables	Negative CTL	Positive CTL
2	Exploratory Screening: 48-hr culture in CDM treatment groups, Cell Painting & HCI (wet lab). Morphological profile data analysis and hit identification (computational)	CDM formulations: Full factorial combinatorial design of 8 growth factors (listed in Figure 1), 2 levels each. 256 total treatment groups (n=4) differentiated by growth factor composition	MSC (Ad98) 1) proliferation and 2) morphology	1) SCM (αMEM + 10% FBS) (n=12) 2) CDBM (n=12)	SCM + 100ng/mL FGF (n=4)
3	Validation Screening: 48-hr culture in CDM hits across 3 MSC donors, Cell Painting & HCI (wet lab). Morphological profile data analysis and hit identification (computational)	1) CDM hits identified from Exploratory Screening (Fig. 2C): Hits A-J (n=4), specific formulations outlined in Fig. 2C. 2) MSC donors: Ad98, BM71, BM115	1) Proliferation and 2) Morphology of each donor: Ad98, BM71, BM115	1) SCM (αMEM + 10% FBS) (n=12) 2) CDBM (n=12)	N/A
4	Initial Expansion: larger scale and extended culture in select CDM hits, assessment of proliferation and immunomodulation at each passage (wet lab)	1) CDM hits selected from Validation Screening (Fig. 3C): Hits A, C, F, G, I, specific formulations outlined in Fig. 3C. 2) Passage: P1, P2, P3	MSC (Ad98) 1) proliferation, 2) IDO activity, 3) sICAM-1 secretion at each passage (P1, P2, and P3)	SCM (αMEM + 10% FBS)	N/A
5	Refinement Screening: 48-hr culture in CDM treatment groups, Cell Painting & HCI (wet lab). Morphological profile data analysis and hit identification (computational)	1) CDM formulations containing FGF, PDGF, LIF, and SCF at different concentrations: Full factorial combinatorial design of 4 growth factors at two levels each (listed in Figure 5A). 16 total treatment groups (n=4) differentiated by growth factor concentrations	MSC (Ad98) 1) proliferation and 2) morphology	1) SCM (αMEM + 10% FBS) (n=12) 2) CDBM (n=12)	Hit G (FGF: 100ng/mL; PDGF: 20ng/mL; LIF: 50 ng/mL; SCF: 20 ng/mL) (n=8)
6	Final Expansion: larger scale culture in select, refined CDM hits; assessment of proliferation and immunomodulation after 1 passage (wet lab)	1) CDM hits selected from Refinement Screening (Fig. 5C): Hit[Low] and Hit[Med] formulations outlined in Fig. 5C.	MSC (Ad98) 1) proliferation, 2) IDO activity, 3) sICAM-1 secretion	SCM (αMEM + 10% FBS)	Hit G (FGF: 100ng/mL; PDGF: 20ng/mL; LIF: 50 ng/mL; SCF: 20 ng/mL)

Table S3. Outline of experiments and experimental groups.

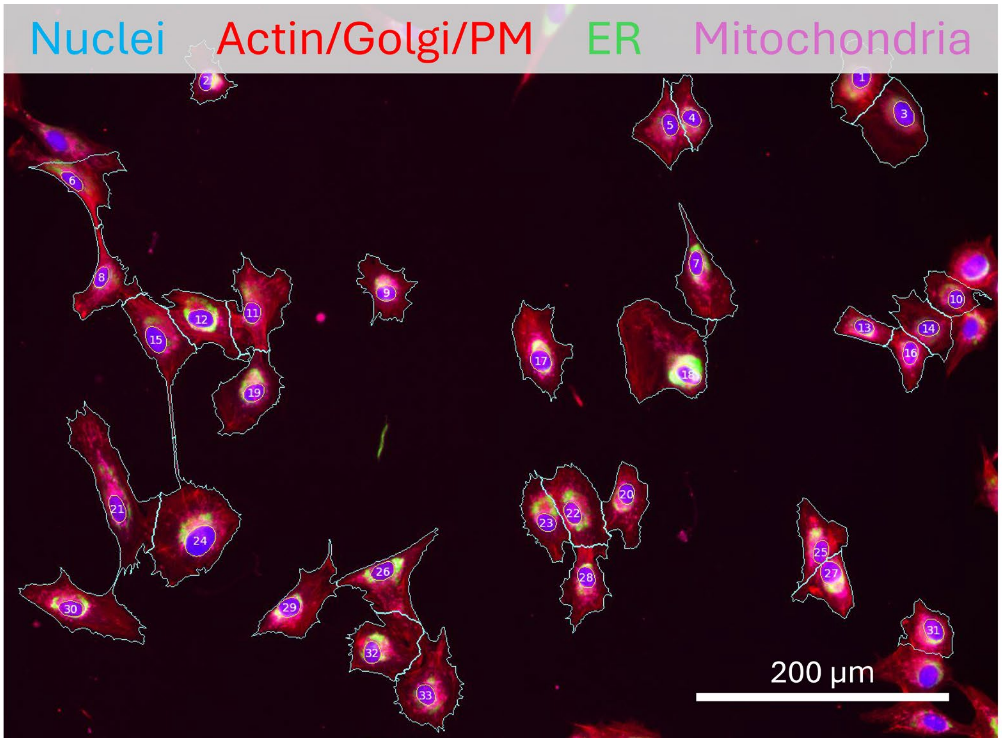

Figure S1: CellProfiler pipeline accurately segments cellular and subcellular components for morphological profiling. Representative color composite image of nuclear (yellow lines) and cellular (cyan lines) segmentation, with number in nucleus representing object count in CellProfiler metadata. PM = plasma membrane, ER = endoplasmic reticulum.

A)

Treatment	Plate #	CDBM *mp*-value	SCM *mp*-value
CDBM	3	0.999	0.004
SCM	3	0	1
Hit J	3	0	0.2
CDBM	4	0.999	0
Hit F	4	0	0
SCM	4	0	0.998
CDBM	5	0.999	0
Hit H	5	0	0
SCM	5	0	1
CDBM	6	0.999	0
Hit D	6	0.001	0.001
SCM	6	0	1
CDBM	9	0.997	0
Hit B	9	0	0.001
SCM	9	0	1
CDBM	10	1	0
Hit G	10	0.001	0.001
SCM	10	0	1
CDBM	12	1	0
Hit I	12	0	0.001
SCM	12	0.745	1
CDBM	13	0.998	0
Hit E	13	0.001	0
SCM	13	0.09	0.997
CDBM	14	0.999	0
Hit C	14	0	0
SCM	14	0.234	0.998
CDBM	15	0.994	0.006
Hit A	15	0.001	0
SCM	15	0.068	1

B)

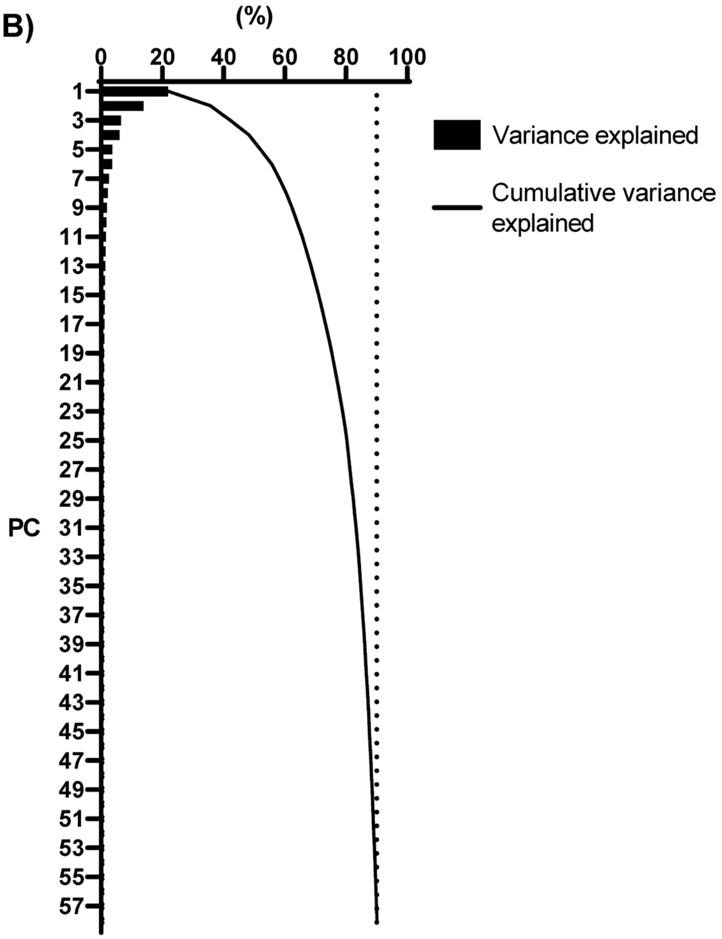

Figure S2: Exploratory HTS mp-value testing highlights phenotypic differences between hits and controls using 58 principal components that represent 90% of the variance in the data. A) mp-values for phenotypic statistical comparisons between each hit and on-plate controls. B) Exploratory HTS PCA cumulative variance plot showing number of PC's that represent 90% of the variance in the data.

A)

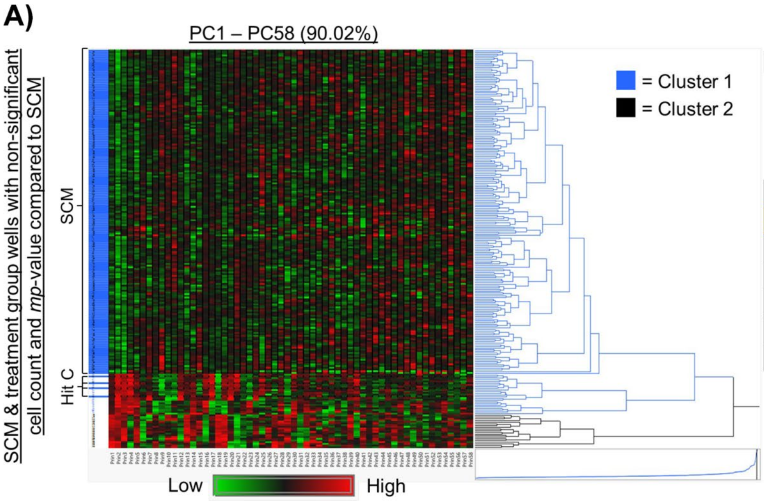

B)

	Cell Count Statistical Comparisons	Morphology Statistical Comparisons
**Treatment**	**SCM adjusted *p* -value**	**SCM *mp* -value**
-TEI-L-A (Hit C)	1	0.2
-TE-----	1	0.171
-T---L-A	1	0.165
-TE----A	0.725872838	0.093
-TE--L-A	0.689338684	0.062
-T-I-LSA	1	0.052

Figure S3: Hierarchical clustering and mp-value testing identify Hit J as CDM formulation most similar to SCM. A) Hierarchical clustering using 58 principal components that represent 90% of the variance in the data of all SCM control wells and treatment groups with non-significant cell counts and mp-values compared to SCM. B) Table showing ranking by mp-value of treatment groups that cluster with SCM. Letters represent growth factors included in the treatment group formulations.

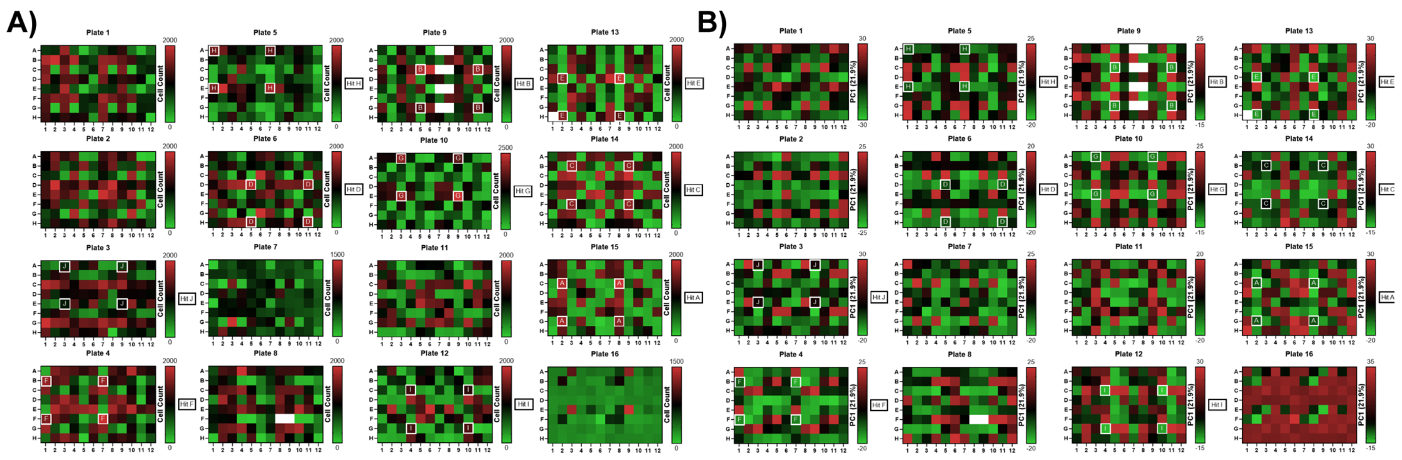

Figure S4: Visual appraisal of exploratory HTS heatmaps serves as quality assessment for hit identification. Heatmaps representing A) cell count and B) PC1. Plates were organized into randomized quadrants of unique conditions, where each condition was repeated across four replicate wells per plate.

A)

Plate 1: AD-MSC RB98		
Treatment	CDBM *mp*-value	SCM *mp*-value
CDBM	1	0
SCM	0	0.997
Hit A	0	0
Hit B	0.001	0
Hit C	0	0
Hit D	0	0
Hit E	0	0
Hit F	0	0
Hit G	0	0.001
Hit H	0	0.001
Hit I	0	0
Hit J	0.002	0

Plate 2: BM-MSC RB71		
Treatment	CDBM *mp*-value	SCM *mp*-value
CDBM	1	0
SCM	0	0.998
Hit A	0	0
Hit B	0	0.001
Hit C	0	0
Hit D	0	0
Hit E	0.001	0.001
Hit F	0	0
Hit G	0	0
Hit H	0	0
Hit I	0.001	0.001
Hit J	0	0

Plate 3: BM-MSC RB115		
Treatment	CDBM *mp*-value	SCM *mp*-value
CDBM	1	0
SCM	0	0.999
Hit A	0	0.001
Hit B	0.001	0
Hit C	0.001	0.001
Hit D	0	0
Hit E	0	0
Hit F	0	0
Hit G	0	0
Hit H	0.001	0.001
Hit I	0.001	0
Hit J	0	0

B)

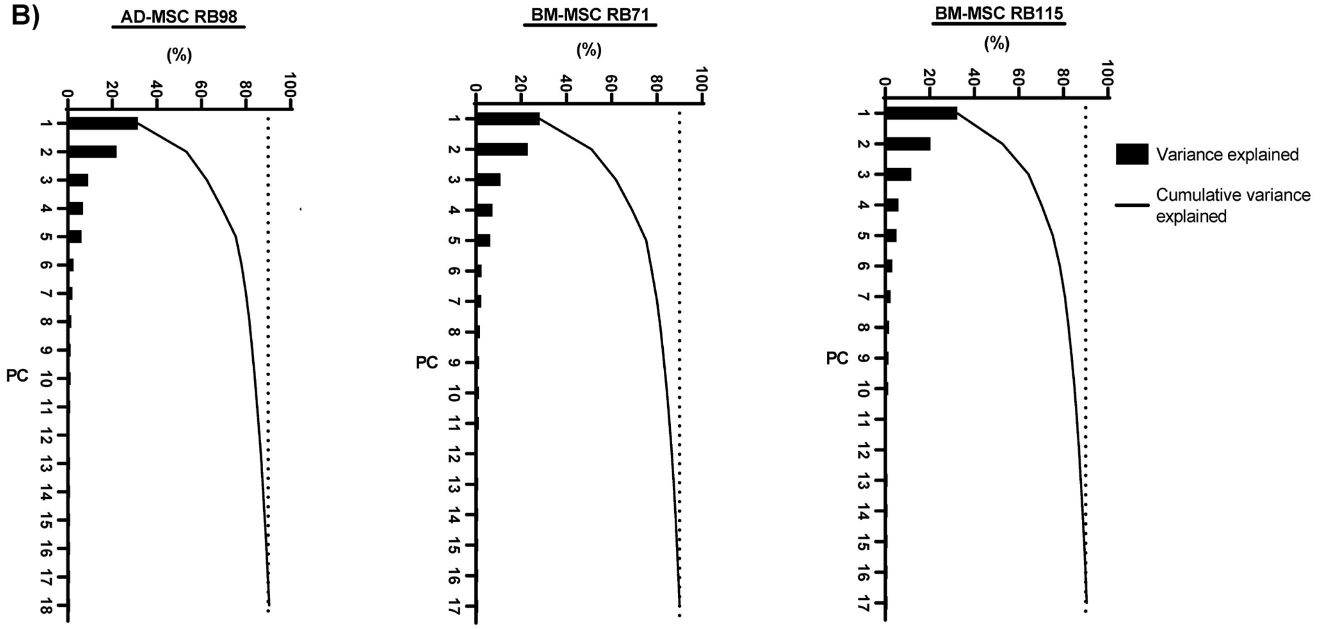

Figure S5: Validation HTS mp-value testing highlights phenotypic differences between hits and controls using 18 principal components that represent 90% of the variance in the data. A) mp-values for phenotypic statistical comparisons between each hit and on-plate controls for each donor. B) Validation HTS PCA cumulative variance plot showing number of PC’s that represent 90% of the variance in the data for each donor.

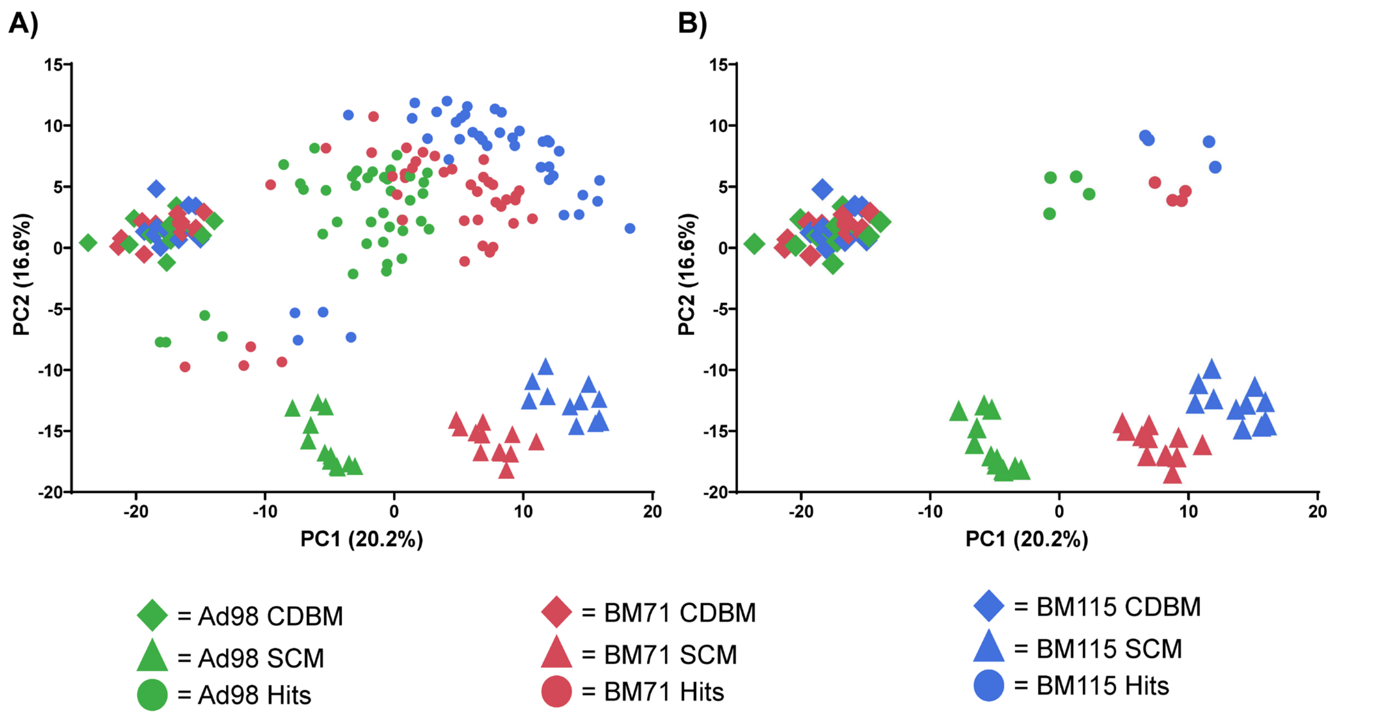

Figure S6: Heterogeneity of morphological responses to different media types persists across multiple MSC donors. PCA plots representing morphological differences across all donors cultured in different media types during validation screen: A) all hits, SCM, CDBM. B) Hit G, SCM, CDBM. Morphological data normalized to CDBM.

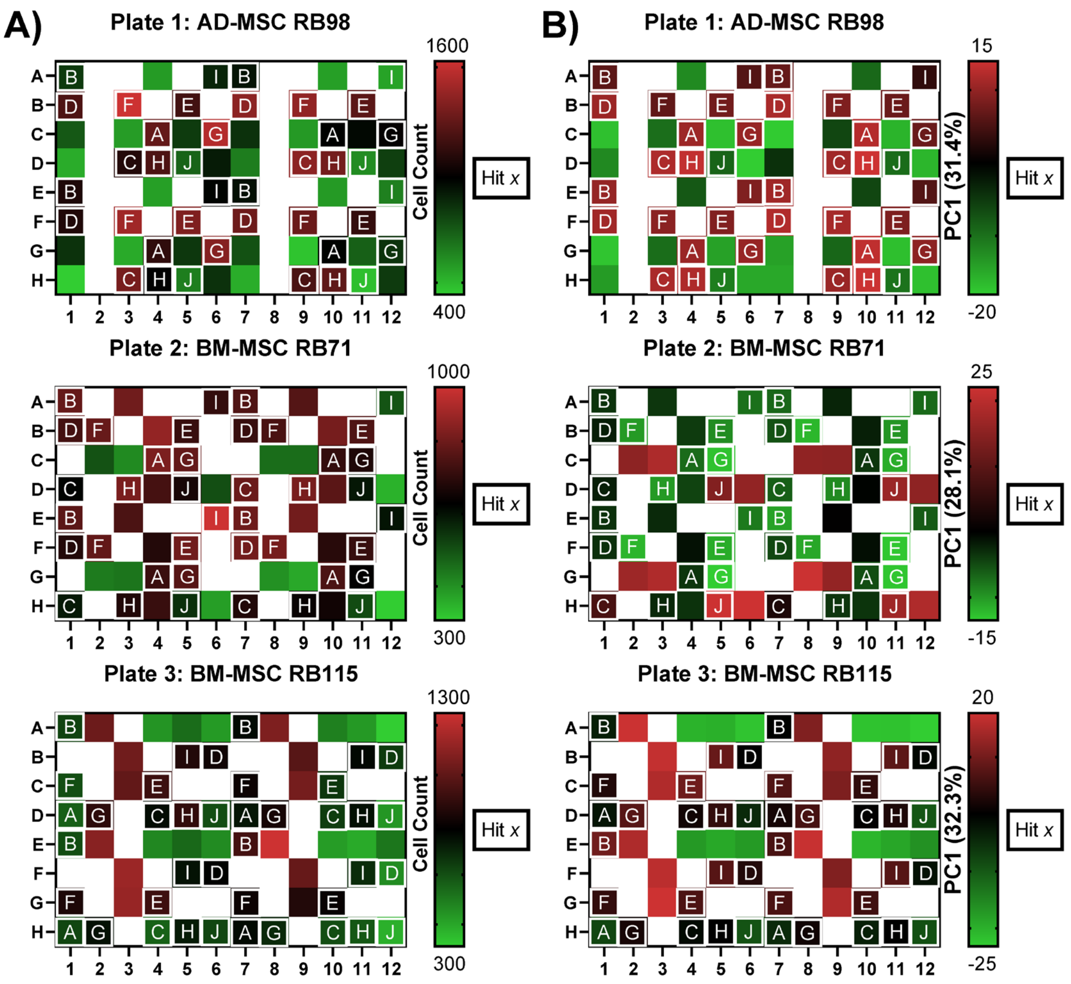

Figure S7: Visual quality assessment of validation HTS heatmaps. Heatmaps representing A) cell count and B) PC1. Plates were organized into randomized quadrants of unique conditions, where each condition was repeated across four replicate wells per plate.

A)

	Morphology Statistical Comparison		
**Treatment**	**SCM *mp*-value**	**CDBM *mp*-value**	**+CTL *mp*-value**
Hit[Low]	0.03	0.003	0.854
Hit[Med]	0.001	0	0.143

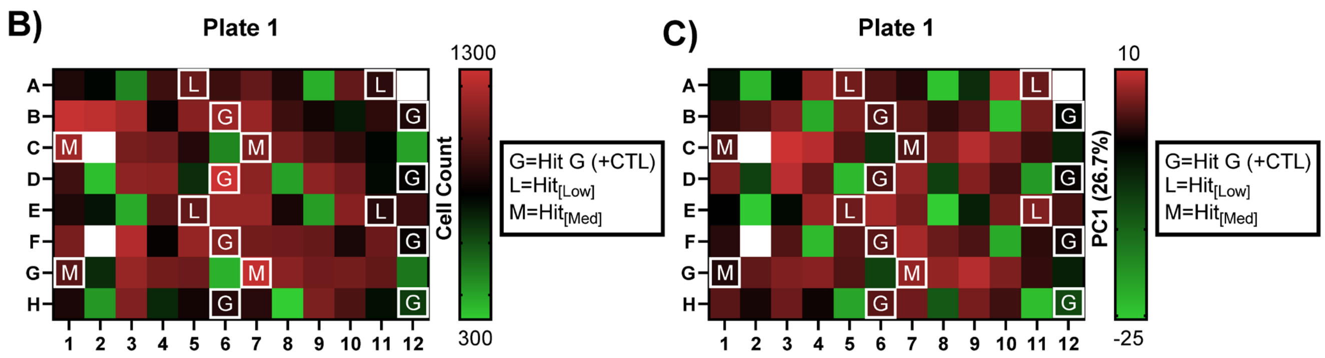

Figure S8: Refinement HTS mp-value testing highlights phenotypic differences between hits and negative controls but similar morphology between hits and +CTL, while heatmaps support replicable biological phenomena of hits. A) mp-values for phenotypic statistical comparisons between each hit and on-plate controls for each donor. Visual refinement HTS quality assessment heatmaps for A) cell count and B) PC1. Plates were organized into randomized quadrants of unique conditions, where each condition was repeated across four replicate wells per plate.

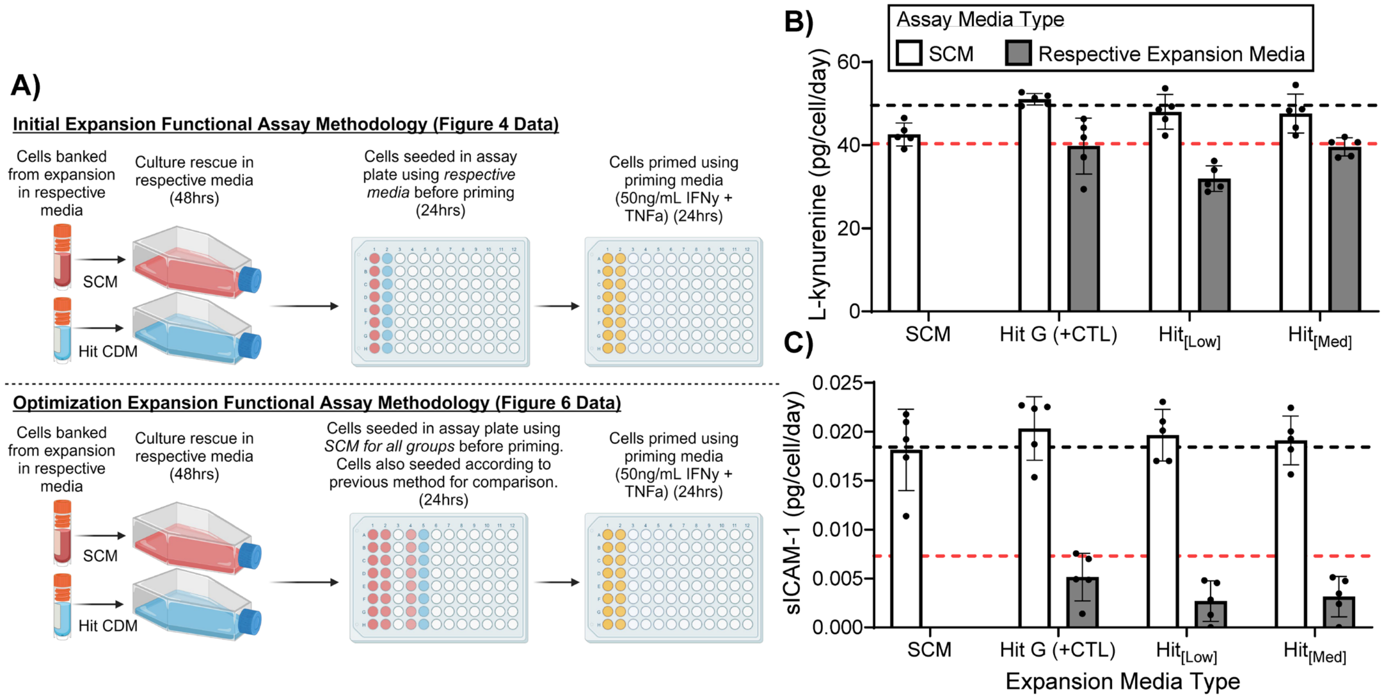

Figure S9: Functional assay methodology impacts MSC response to priming. A) Schematic representing differences in functional assay methodology tested after refinement screen expansion. B) IDO activity versus expansion media type and media type used for seeding in assay plate. Black dashed line = L-kynurenine (pg/cell/day) after P1 of initial expansion for MSCs grown in SCM (Fig. 4E). Red dashed line = L-kynurenine (pg/cell/day) after P1 of initial expansion for MSCs grown in Hit G (+CTL) (Fig. 4E). C) sICAM-1 secretion versus expansion media type and media type used for seeding in assay plate. Black dashed line = sICAM-1 (pg/cell/day) after P1 of initial expansion for MSCs grown in SCM (Fig. 4E). Red dashed line = sICAM-1 (pg/cell/day) after P1 of initial expansion for MSCs grown in Hit G (+CTL) (Fig. 4E).
